# Evaluation of immunization coverage within the Expanded Program on Immunization in Kita Circle, Mali: a cross-sectional survey

**DOI:** 10.1186/1472-698X-9-S1-S13

**Published:** 2009-10-14

**Authors:** Abdel Karim Koumaré, Drissa Traore, Fatouma Haidara, Filifing Sissoko, Issa Traoré, Sékou Dramé, Karim Sangaré, Karim Diakité, Bréhima Coulibaly, Birama Togola, Aguissa Maïga

**Affiliations:** 1 Faculté de Médecine de Pharmacie et d'Odonto Stomatologie, Université de Bamako, Bamako, Mali; 2 Institut Africain de Formation en Pédagogie, Recherche et Evaluation en Sciences de la Santé (IAFPRESS) - Quartier du Fleuve - Bamako, BP 05 Koulouba, Mali; 3 Centre de Santé de Référence de la commune V, Bamako, Mali; 4 Centre de Santé de Réference de la commune V du cercle de Kita, Mali; 5 Direction Régionale de la Santé de Kayes, Mali

## Abstract

**Background:**

In 1986, the Government of Mali launched its Expanded Program on Immunization (EPI) with the goal of vaccinating, within five years, 80% of all children under the age of five against six target diseases: diphtheria, tetanus, pertussis, poliomyelitis, tuberculosis, and measles. The Demographic and Health Survey carried out in 2001 revealed that, in Kita Circle, in the Kayes region, only 13% of children aged 12 to 23 months had received all the EPI vaccinations. A priority program was implemented in 2003 by the Regional Health Department in Kayes to improve EPI immunization coverage in this area.

**Methods:**

A cross-sectional survey using Henderson's method (following the method used by the Demographic and Health Surveys) was carried out in July 2006 to determine the level of vaccination coverage among children aged 12 to 23 months in Kita Circle, after implementation of the priority program. Both vaccination cards and mothers' declarations (in cases where the mother cannot make the declaration, it is made by the person responsible for the child) were used to determine coverage.

**Results:**

According to the vaccination cards, 59.9% [CI 95% (54.7-64.8)] of the children were fully vaccinated, while according to the mothers' declarations the rate was 74.1% [CI 95% (69.3-78.4)]. The drop-out rate between DTCP1 and DTCP3 was 5.5%, according to the vaccination cards. The rate of immunization coverage was higher among children whose mothers had received the anti-tetanus vaccine [OR = 2.1, CI 95% (1.44-3.28)]. However, our study found no difference associated with parents' knowledge about EPI diseases, distance from the health centre, or socio-economic status. Lack of information was one reason given for children not being vaccinated against the six EPI diseases.

**Conclusion:**

Three years after the implementation of the priority program (which included decentralization, the active search for missing children, and deployment of health personnel, material and financial resources), our evaluation of the vaccination coverage rates shows that there is improvement in the EPI immunization coverage rate in Kita Circle. The design of our study did not, however, enable us to determine the extent to which different aspects of the program contributed to this increase in coverage. Efforts should nevertheless be continued, in order to reach the goal of 80% immunization coverage.

**Abstract in French:**

See the full article online for a translation of this abstract in French.

## Abstract in French

See Additional file 1 for a translation of the abstract to this article in French.

## Background

Vaccination is the most effective means of combating disease. Vaccines exist for a great many dangerous infectious diseases. The introduction of vaccines, particularly among children, has led to significant reductions in morbidity and mortality from these diseases, thereby lowering the infant mortality rate. However, in sub-Saharan Africa, despite the availability of these vaccines and efforts on the part of governments and their partners to make them accessible, the mortality rate for children under the age of five remains among the highest in the world [1]. In 1974, the World Health Organization (WHO) launched the Expanded Program on Immunization (EPI) to make vaccines available to all children worldwide [2]. In Mali the government launched its EPI in 1986 with the goal of vaccinating, within five years, 80% of all children under the age of five against six target diseases: diphtheria, tetanus, pertussis, poliomyelitis, tuberculosis, and measles. After many years, this goal is far from being reached [3,4], and in 2006, the infant mortality rate was 119 per 1 000 and child mortality, 217 per 1,000 -- both rates still among the highest in the world [5]. Fifteen years after the EPI's inauguration, the 2001 Demographic and Health Survey (DHS-III) [6] revealed that, according to vaccination records or mothers' declarations, only 32% of children between the ages of 12 and 23 months had received all the EPI vaccinations. This poor performance was even more striking in the Kayes region in the northwest area of the country, including Kita Circle (a circle being an administrative district), where only 13.6% of children aged 12 to 23 months had been fully vaccinated.

The DTCP1 to DTCP3 drop-out rate in the Kayes region was among the highest in Mali (60%); in other regions of the country the rate ranged from 12% to 50% (DHS-II, DHS-III) [7]. The very great disparity between the routine data (which allows health personnel of the Kayes region to plan EPI activities based on "coverage rates" of around 70%) and DHS data (with rates of fully vaccinated children being under 15%, without taking into consideration whether they were correctly vaccinated), must have shaken the convictions of health personnel in the Kayes region, and especially those of the regional authorities. In fact, the administrative authorities based themselves largely on the results from the National Immunization Days (NID), which have a rate of coverage of more than 90% [7]. For these reasons, in 2003, the Regional Department of Health in Kayes developed a priority program to improve the EPI's rate of immunization coverage.

This priority program had seven main components:

1. The creation of community health centres (CSCom - Centres de santé communautaire) for each 5 000 habitants. Each CSCom was provided with the human and material resources required to carry out vaccination sessions within a radius of 15 km. Before the implementation of this program, vaccination units at times had to vaccinate children within a radius of 100 km.

2. The creation of a health committee within each village. Each committee was composed of two villagers (one male and one female) who were called "intermediaries". They received training on the EPI (targeted diseases and vaccinations schedule) and were provided with a register allowing them to record all pregnant women in the village, all births, all children between the ages of 0 and 5, and the vaccination calendars for these children. These intermediaries were in regular contact with vaccination teams, which allowed them to know the dates of vaccination sessions and thereby inform villagers in advance. Upon the arrival of vaccinators, the intermediaries would accompany them in the village, and would thereby know, at the end of the vaccination session, which children were missing. When vaccinators left, they would enquire about the reasons why children had missed the vaccination session and plan for them to attend the following one. This was known as the active search for missing children.

3. The purchase of a motorcycle, its maintenance, and the purchase of fuel for the vaccination team.

4. The purchase of a 4 × 4 vehicle, its maintenance, and the purchase of fuel for the supervision team.

5. The purchase of equipment for, and maintenance of, the cold chain.

6. The purchase of vaccination cards.

7. The regular payment of per diems for the training and supervision sessions.

This priority program was mainly financed by the Global Alliance for Vaccines and Immunization (GAVI). Here, we report on the results of an evaluation of the vaccination coverage rate three years after the implementation of the priority program in the Kayes region. We did not, however, evaluate the priority program itself.

## Methods

### Location of the study

The study took place in Kita Circle, which was selected as representative of the Kayes region. Kita Circle is located in the southwest part of the Kayes region and has a land area of 35 250 km^2^. It has an estimated population of 338 551 people distributed among 330 villages regrouped into 33 communes, of which 2 are urban and 31 rural. The circle is served by a railway line linking it to Bamako and Kayes, at distances of 186 and 307 km, respectively. At the time of the study, the circle had only regional roads and rural tracks; there were no asphalt roads. In addition to its connection to the telephone network, the circle had seven local radio stations as well as traditional methods of communication. The national television covered approximately 30% of the circle's territory.

As in the rest of the country, the EPI vaccines are administered according to the calendar set out by WHO [8]: Polio 0 and BCG in the first 15 days after birth; DTCP1, DTCP2, and DTCP3 at, respectively, 6, 10, and 14 weeks after birth; measles and yellow fever, at nine months of age. The viral hepatitis B and *haemophilus influenzae *type B vaccines, introduced more recently into Mali's EPI, are administered in combination and at the same time as DTCP1, DTCP2, and DTCP3.

### Outline of the study

The survey was carried out in July 2006 in three health areas of the circle (Djidjan, Fladougou, and Kasaro) and was focused on children between the ages of 12 and 23 months. The WHO protocol developed by Henderson [9] for evaluating EPI immunization coverage was used; the same method was used in the DHS-III in 2001. With this method, to be accurate within 10% with a margin of error of 5% would require surveying 210 children per health area. For greater accuracy, we surveyed around 250 children per health area. In each health area, children were selected using a random sampling; sample size was proportional to the population size. For each child selected, information on the vaccination card and statements made by the person responsible for the child during an individual guided interview were noted. There is a discrepancy between data taken only from the vaccination cards and those based also on the mothers' statements. Thus, if we exclude the mother as a source of information, 4% of the children are considered to be only partially, rather than fully, vaccinated. The rate of fully vaccinated children thus becomes 26% instead of 30% [6].

Mothers' statements are subject to bias because they:

• might be subjected to historical biases;

• do not allow us to know if the vaccination was carried out at the right time;

• and therefore, do not allow us to properly study dropouts.

In addition, illiterate mothers often cannot tell the difference between the EPI vaccinations and those of the NID, nor the differences among the various antigens.

Nevertheless, including them helps minimize the underestimation of immunization coverage that occurs because of lost vaccination cards. Given the biases related to the choice of one method over the other, it was decided to use the same definition as in the official reports of the DHS, which use both information from vaccination cards and mothers' declarations. Children were eligible if they had been residents for at least six months and if their parents consented to their participation in the study. Thirteen pairs of trained supervisors who spoke French well and at least one local language (Malinke, Khassonké, Bambara, Soninké, and Peul) carried out the interviews with the mothers using a questionnaire that was validated after pre-testing. Supervision was ensured by three physicians.

For each child, in addition to the data on the vaccination card, the following information was gathered: i) parents' knowledge about the diseases covered by the EPI; ii) distance from the health centre; iii) prenatal consultations and whether the mother was vaccinated against tetanus during pregnancy; and iv) socio-economic status, determined by the number of meals per day and whether the family possessed a radio and/or a television. Parents were also questioned about the reasons for non-vaccination or for dropping out between DTCP1 and DTCP3 (child having received DTCP1 but not DTCP3). Data collected on the questionnaires were coded and copied into Epi Info version 6 [10]. Analyses were carried out in SPSS version 12.0. The rate of immunization coverage was estimated as the proportion of children who had been fully vaccinated against the six diseases of the EPI, with a confidence level of 95% using the method of Fleiss [11] and a cluster effect of two [12]. Pearson's chi-square test was used to compare the proportions. Ratings ratios were calculated to assess the association between full immunization coverage and the other dimensions studied (parents' knowledge of EPI diseases, distance from health centre, prenatal consultations and mother's vaccination against tetanus during pregnancy, socio-economic status). A child is considered fully vaccinated if all EPI vaccines against the six targeted diseases were received.

## Results

The numbers of children surveyed in each health area were: 252 in Djidjan, 250 in Fladougou, and 248 in Kasaro, for a total of 750 children, of whom 378 (50.4%) were male.

### Proportion of children vaccinated, by antigen

Immunization coverages by antigen and by health area are presented in Table 1. Total immunization coverages for BCG, DTCP1, DTCP2, and DTCP3 were above 90%, while coverage for measles was 70.5%.

**Table 1:** Proportion of children having received the various EPI antigens according to vaccination cards, by health area (2006).

	Djidjan (n= 252)	Fladougou (n= 250)	Kasaro (n= 248)	Total (n = 750)
BCG	92.1	92.0	91.5	91.9
DTCP1	96.4	98.4	92.3	95.7
DTCP2	95.6	93.2	90.7	93.2
DTCP3	94.1	92.4	87.1	91.2
Measles	69.4	74.8	67.3	70.5

### Proportion of children fully vaccinated

The immunization coverage levels observed are presented in Table 2. The rate of children fully vaccinated according to the mothers' declarations was highest in the Djidjan health area (78.2%), followed by Fladougou (76.4%) and Kasaro (67.7%). The rate of fully vaccinated children according to vaccination cards was highest in the Fladougou health area (63.2%), followed by Djidjan (58.3%) and Kasaro (58.1%). In the three health areas combined, the rate of fully vaccinated children according to the statements of those responsible for them was higher, at 74.1% [CI 95% (69.3-78.4)], than it was according to the vaccination cards, at 59.9% [CI 95% (54.7-64.8)].

**Table 2:** Proportion of fully vaccinated children according to the mothers' declarations and the vaccination cards, by health area (2006).

	Statements			Vaccination card		
		
Health area	n	%	CI 95%	n	%	CI 95%
Djidjan	252	78.2	69.8-84.8	252	58.3	49.2-66.9
Fladougou	250	76.4	70.5-81.4	250	63.2	54.1-71.5
Kasaro	248	67.7	58.7-75.7	248	58.1	48.9-66.8
Total	750	74.1	69.3-78.4	750	59.9	54.7-64.8

### DTCP1 to DTCP3 drop-out rate

If we rely on the vaccination cards held by the families, the rate of drop-out between DTCP1 and DTCP3 was significantly lower in Djidjan, at 2.8%, than in Fladougou (7.6%) and Kasaro (6%).

### Factors associated with full vaccination

The associations between full vaccination and the factors studied are presented in Table 3. Among the factors studied, only the mother's vaccination status during pregnancy was associated with full vaccination of the child [OR = 2.18, CI 95% (1.44-3.28)]. On the other hand, there was no link between full vaccination and the other factors, particularly the mother's education, prena-tal consultations, parents' knowledge about the EPI diseases, the child's sex, distance from the health centre, or socio-economic status.

**Table 3:** Vaccination status and associated factors (2006).

		% Fully vaccinated		
Factors		n	children	p
Mother's anti-tetanus	Yes	627	63.0	<0.001
vaccine	No	123	43.9	
				
Sex	Male	378	59.8	0.9
	Female	372	59.9	
				
Mother's education	Yes	111	65.8	0.16
	No	639	58.8	
				
Prenatal consultations	Yes	537	61.8	0.08
	No	213	54.9	
				
Knowledge about EPI diseases	Yes	57	61.4	0.8
	No	693	59.7	
				
Meals per day	1	82	56.1	0.46
	2-3	668	60.3	
				
Possession of radio	Yes	641	59.9	0.45
	No	109	59.6	
				
Possession of television	Yes	180	62.2	0.45
	No	570	59.1	
				
Distance from health centre	<6 km	330	45.9	0.35
	6-14 km	198	26.3	
	>14 km	222	27.8	

### Reasons for non-vaccination and for dropping out according to those responsible for the children

**Figure 1:**
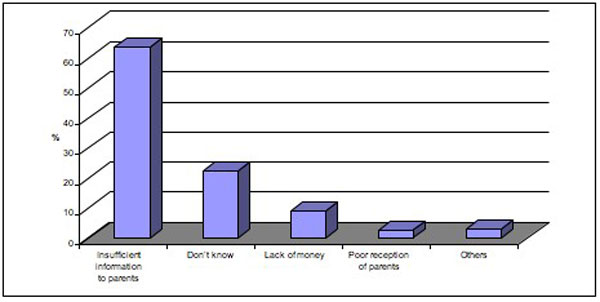
**Reasons for child's non-vaccination or dropping out, according to the mothers' declarations**.

The reasons for non-vaccination and for dropping out, according to those responsible for the children, are presented in Figure 1. The reason most often mentioned was insufficient information (63.3% of respondents), followed by a lack of money to buy the card or for travel (8.9%). Parents' refusal was mentioned by 4% of those responsible for children to explain non-vaccination or dropping out, while 2.4% cited unwelcoming reception and an overly long wait time.

## Discussion

As shown in Tables 1 and 2, the rate of fully vaccinated children according to declarations of those responsible for them improved considerably between 2001 and 2006. Indeed, according to our study, this rate went from 13.6% in 2001 in the Kayes region to 74% in the three health areas of Kita. The studies carried out as part of the DHS-III in 2005 in the six communes of Bamako show rates of fully vaccinated children, according to the declarations of the person responsible for the child, ranging from 76.2% to 86.5% [13-18]. In Kita Circle, this increase in coverage can in part be explained by the fact that before 2003, a large part of the population (around 15%) lived between 15 and 100 km of the nearest vaccination centre, resulting in various transportation issues. Now, however, with the implementation of the priority program, the population lives within 15 km of a CSCom, and thus of vaccination centres. At the same time, it is also postulated that other aspects of the priority program, including the active search for missing children, contributed to this increase.

The rate of fully vaccinated children according to vaccination cards has also improved markedly between 2001 and 2006: going from 5% in 2001 in the Kayes region (according to the DHS-III) to 60% (according to our study). This rate was between 60% and 72.6% in the six communes of Bamako in 2005 [13-17].

According to the DHS-III in 2001 [8], immunization coverage was twice as great in urban settings, particularly in Bamako (52%), as in rural ones (24%). Our study, however, shows an increase in coverage rates in the three health areas of Kita Circle (all rural areas) to levels similar to those of Bamako. There is, therefore, a reduction of inequities in vaccination coverage between the rural zone of Kita and the capital city. Again, this increase in the vaccination coverage rate in the three health areas of Kita is likely linked to the priority program formulated and implemented in Kita Circle and financed mainly by GAVI. The lower rate for measles coverage is likely due to problems in the stock supply of measles vaccines in the region a few months prior to this study, as explained by the doctor in charge of the region.

Our study has also demonstrated that the probability that children will be vaccinated rises when the mother herself is vaccinated against tetanus, a finding that mirrors those of other authors [19,20]. We did not find any significant differences in rates of immunization coverage that could be related to the sex of the child or to the socio-economic status of the family, as reported in earlier studies [19,20]. In this case, the absence of any difference could be explained by the presence, as part of the priority program, of two intermediaries in each village who follow up on children who do not attend a vaccination session and plan for them to attend the following one, regardless of the children's sex or socio-economic status. It should be noted, however, that the fact that socio-economic status did not have any influence on vaccination coverage rates might be due to the inaccuracy of the indicators we used to measure socio-economic status.

While our initial hypothesis was that drop out rates were influenced by lack of money to pay for vaccination cards and poor reception by health personnel, the study showed that parents mention those reasons in less than 10% of drop-out cases, while they blame insufficient information 60% of the time. The observation that persons responsible for children most often mentioned insufficient information as the primary reason for non-vaccination or dropping out is not surprising and confirms the work of other authors [18,19,21,22]. However, these declarations contradict the other results of our study, which found that the level of knowledge of the EPI did not influence the vaccination coverage rate. These contradictory findings deserve a more detailed qualitative study in order to determine the real reasons behind non-vaccination or dropping out.

Figure 1 demonstrates that health personnel were held accountable for non-vaccination or dropping out in 2.4% of the cases in this study, particularly because of unwelcoming reception or overly long wait times. In other studies, the main reason given for dropping out was the long wait time [13-17]. In our questionnaire, however, we did not differentiate between reasons for non-vaccination and reasons for dropping out.

All the reasons provided for non-vaccination or dropping out underscore the need to give priority to providing information and raising the awareness of populations, even if earlier studies have demonstrated the limited efficacy of Information, Education, and Communication (IEC) sessions in health facilities [19,20]. However, the absence of any significant differences with respect to mother's education, prenatal consultations, parents' knowledge about the EPI diseases, child's sex, distance from the health centre, or socio-economic status is a reflection of the limitations of our study. These include: the insufficient strength of our sample; the lack of control groups; and the lack of an experimental design to actually evaluate the priority program.

If insufficient information is indeed confirmed to be a key factor in other contexts as well, further questions to be addressed by other studies could include:

• What are the factors that influence vaccination coverage in areas with active search for missing children versus those areas without it?

• What are the best strategies for raising awareness among illiterate people, to persuade them to have their children vaccinated without the need for the active search for missing children?

Among the eight circles of the Kayes region of Mali, our study looked only at Kita Circle. We selected Kita Circle because it had the lowest rates of immunization coverage in a region that, itself, had the lowest immunization coverage in the country. Even if there is no reason *a priori *to believe that immunization coverage in the other circles would be lower than in Kita Circle, it would be interesting to confirm this by an evaluation in one or more of these circles.

The increase in immunization coverage from 13.6% in 2001 in the whole region of Kayes to 74% in our study three years after implementation of the priority program demonstrates that it is possible, by using appropriate strategies, to significantly improve immunization coverage in the country. In this case, it would appear that decentralization of health activities has indeed contributed to an increase in coverage, but this needs to be coupled with the mobilization of appropriate resources (as was the case here with the support of GAVI) if objectives are to be attained.

## Conclusion

Three years after the implementation of the priority program (which included decentralization, the active search for missing children, and deployment of health personnel, material and financial resources), our evaluation of the vaccination coverage rates shows that there is improvement in the EPI immunization coverage rate in Kita Circle. The design of our study did not, however, enable us to determine the extent to which different aspects of the program contributed to this increase in coverage. Efforts should nevertheless be continued, in order to reach the goal of 80% immunization coverage, and, as the study identified, notably through better information to parents.

## List of abbreviations used

DHS-II: Demographic and Health Survey, 2nd edition; DTCP: Diphtheria, tetanus, pertussis, poliomyelitis; DHS-III: Demographic and Health Survey, 3rd edition; IEC: Information, Education and Communication; WHO: World Health Organization; EPI: Expanded Program of Immunization; TV: Television; CI: Confidence interval; OR: Odds ratios; BCG: Bacillus Calmette-Guérin; GAVI: Global Alliance of Vaccines and Immunization.

## Competing interests

They authors declare they have no competing interests.

## Authors' contributions

AKK contributed to the design of the study, supervision of the surveys, and the writing of the manuscript; FS contributed to the supervision of the survey. IT, SD, KS, and KD participated in supervising the survey and writing the report. AM participated in writing the report. DT, FH, and AD contributed to writing the analysis and the manuscript.

## Supplementary Material

Additional file 1 Abstract in French. Click here for file
