# Supplementary material for: Evaluation of immunization coverage within the Expanded Program on Immunization in Kita Circle, Mali: a cross-sectional survey
Source: BMC Int Health Hum Rights. 2009 Oct 14;9(Suppl 1):S13. doi: 10.1186/1472-698X-9-S1-S13 (PMC3226232; doi:10.1186/1472-698X-9-S1-S13)
Supplement: Additional file 1 — Abstract in French. [file 1472-698X-9-S1-S13-S1.pdf]

Évaluation de la couverture vaccinale dans le cadre du Programme Élargi de Vaccination dans le cercle de Kita, Mali: une étude transversale

Abdel Karim Koumaré, Drissa Traore, Fatouma Haidara, Filifing Sissoko, Issa Traoré, Sékou Dramé, Karim Sangaré, Karim Diakité, Bréhima Coulibaly, Birama Togola, Aguissa Maïga

Résumé

Problématique

Le Programme Élargi de Vaccination (PEV), lancé au Mali en 1986, prévoyait d'atteindre en cinq ans une couverture vaccinale de 80% contre six maladies cibles (diphtérie, tétanos, coqueluche, poliomyélite, rougeole et tuberculose). L'Enquête Démographique et de Santé (EDS) de 2001 révèle qu'à peine 13% des enfants âgés de 12 à 23 mois du cercle de Kita, dans la région de Kayes, avaient été complètement vaccinés contre les six maladies du PEV. Un programme prioritaire y a été introduit en 2003 par le Département sanitaire régional afin d'améliorer la couverture vaccinale dans la région.

Méthodes

L'étude repose sur une enquête de couverture vaccinale réalisée en juillet 2006 auprès des enfants âgés de 12 à 23 mois dans le cercle de Kita. Elle repose sur la méthode d'Henderson, utilisée notamment pour les EDS. La couverture vaccinale a été estimée à partir des carnets de vaccination et des déclarations des mères (dans le cas où la mère ne pourrait pas faire la déclaration, elle est faite par la personne en charge de l'enfant).

Résultats

La couverture vaccinale complète estimée est de 59.9% (54.7–64.8) sur la base des carnets de vaccination, et de 74% si on se fie aux déclarations des mères (69.3–78.4). Le taux de déperdition entre DTCP1 et DTCP3 est en nette diminution, à 5.5% selon les cartes de vaccination. La couverture est plus élevée chez les enfants dont la mère avait reçu une vaccination antitétanique [OR = 2.1, (1.44–3.28)]. Les connaissances des parents des maladies évitables par la vaccination, leur niveau socio-économique ou la distance entre le domicile et le centre de santé ne sont pas associés significativement à la couverture. Le manque d'information est par contre une des raisons évoquées par les parents pour expliquer pourquoi les enfants ne sont pas vaccinés contre les six maladies du PEV.

Conclusions

Trois ans après l'implantation du programme (qui incluait la décentralisation, la recherche active des enfants non vaccinés, et le déploiement de personnel de santé et de ressources matérielles et financières), notre évaluation de la couverture vaccinale démontre une nette progression de la couverture vaccinale du PEV dans le cercle de Kita. Le format de l'étude ne nous a par contre pas permis de déterminer dans quelle mesure les différents aspects du programme prioritaire ont contribué à cette augmentation de la couverture vaccinale. Les autorités locales doivent tout de même maintenir leurs efforts pour atteindre les objectifs nationaux de couverture qui ne sont pas encore rejoints.
